# Prolonged oral antimicrobial administration prevents doxorubicin-induced loss of active intestinal stem cells

**DOI:** 10.1080/19490976.2021.2018898

**Published:** 2022-01-11

**Authors:** Breanna J Sheahan, Casey M Theriot, Jocsa E. Cortes, Christopher M Dekaney

**Affiliations:** a Molecular Biomedical Sciences, College of Veterinary Medicine, NC State University, Raleigh, NC USA; b Department of Pharmacology and Cancer Biology, Duke University, Durham, Nc USA; c Population Health and Pathobiology, College of Veterinary Medicine, NC State University, Raleigh, NC USA

**Keywords:** Microbiota, intestinal stem cells, doxorubicin, antibiotics, antimicrobials, DNA damage, germ free, injury, small intestine

## Abstract

Acute intestinal mucositis is a common off-target effect of chemotherapy, leading to co-morbidities such as vomiting, diarrhea, sepsis, and death. We previously demonstrated that the presence of enteric bacteria modulates the extent of jejunal epithelial damage induced by doxorubicin (DXR) in mice. Despite conventional thinking of the crypt as a sterile environment, recent evidence suggests that bacterial signaling influences aISC function. In this study, we labeled aISCs using transgenic *Lgr5*-driven fluorescence or with immunostaining for OLFM4. We examined the effect of DXR in both germ free (GF) mice and mice depleted of microbiota using an established antimicrobial treatment protocol (AMBx). We found differences in DXR-induced loss of aISCs between GF mice and mice treated with AMBx. aISCs were decreased after DXR in GF mice, whereas AMBx mice retained aISC expression after DXR. Neither group of mice exhibited an inflammatory response to DXR, suggesting the difference in aISC retention was not due to differences in local tissue inflammation. Therefore, we suspected that there was a protective microbial signal present in the AMBx mice that was not present in the GF mice. 16S rRNA sequencing of jejunal luminal contents demonstrated that AMBx altered the fecal and jejunal microbiota. In the jejunal contents, AMBx mice had increased abundance of *Ureaplasma* and *Burkholderia*. These results suggest pro-survival signaling from microbiota in AMBx-treated mice to the aISCs, and that this signaling maintains aISCs in the face of chemotherapeutic injury. Manipulation of the enteric microbiota presents a therapeutic target for reducing the severity of chemotherapy-associated mucositis.

## Introduction

Acute intestinal mucositis is a common off-target effect of chemotherapy, leading to co-morbidities such as vomiting, diarrhea, sepsis, and death.^1^ These complications can arise secondary to loss of active intestinal stem cells (aISCs), which live in the crypt base and produce all intestinal epithelial cells.^2^ These cells are identified by high expression of olfactomedin 4 (Olfm4) and leucine-rich repeat-containing G-protein coupled receptor 5 (Lgr5).^3^ aISCs live in close proximity to the gut microflora. The intimate association of aISCs and gut microbiota suggests that microbial signals may influence aISCs’ responses to damage.

We have previously demonstrated that the presence of enteric bacteria modulates the extent of jejunal epithelial damage induced by chemotherapy in mice.^4^ Doxorubicin (DXR), a commonly used chemotherapeutic agent, induces a reliable sequence of apoptosis and impaired proliferation followed by epithelial regeneration.^1^ However, under germ free (GF) or microbial depletion conditions, the impaired proliferation and immune cell infiltration does not occur.^4,5^ Recent work from our lab demonstrates acute loss of aISCs in response to doxorubicin in conventionally raised (CONV) mice with wild-type microbiota.^6^

Despite conventional thinking of the crypt as a sterile environment, recent evidence suggests that bacterial signaling influences aISC function.^7–9^ Bacterial-derived lactate signals by binding to a receptor present on both Paneth cells and subepithelial cells to drive proliferative Wnt signaling in aISCs.^7^ It has recently been shown that activation of aISC-associated innate immune receptor nucleotide-binding oligomerization domain-containing protein 2 (NOD2) by muramyl dipeptide (MDP) protects aISCs from excessive reactive oxygen species (ROS) damage.^9,10^ Damage models where NOD2 activation was protective included DXR and irradiation *in vivo* and *in vitro*.^9,10^ In contrast, lipopolysaccharide (LPS) binding to toll-like receptor 4 (TLR4) on colonic stem cells increases apoptosis in these stem cells and impairs crypt proliferation.^11,12^ Therefore, potential known mechanisms of bacterial-derived protection of stem cells from injury include 1) increased crypt lactate or increased MDP-NOD2 signaling to aISCs, and 2) decreased LPS-TLR4 signaling to aISCs. We had previously observed reduced epithelial damage secondary to DXR in GF and microbial depletion models. Thus, we theorized that manipulating bacterial signaling to aISCs by removal or depletion of enteric microbiota would enhance aISC survival after DXR-induced damage.

In this study, we labeled aISCs using transgenic *Lgr5*-driven fluorescence or with immunostaining for OLFM4. We initially examined the effect of DXR in both germ free mice and mice depleted of microbiota using an established protocol.^13,14^ We found differences in DXR-induced loss of aISCs between GF mice and mice treated with broad-spectrum antimicrobials (AMBx). aISCs were decreased after DXR in GF mice, whereas AMBx mice retained aISC expression after DXR. Neither group of mice exhibited an inflammatory response to DXR, suggesting the difference in aISC retention was not due to differences in local tissue inflammation. Therefore, we suspected that there was a protective microbial signal present in the AMBx mice that was not present in the GF mice. 16S rRNA sequencing of jejunal luminal contents demonstrated that AMBx altered the fecal and jejunal microbiota. In the jejunal contents, AMBx mice had increased abundance of two genera within the Firmicutes and Proteobacteria phyla: *Ureaplasma* and *Burkholderia*, respectively. Future studies should assess whether these genera are the cause of the aISC retention. These results suggest pro-survival signaling from microbiota in AMBx-treated mice to the aISCs, and that this signaling maintains aISCs in the face of chemotherapeutic injury. Manipulation of the enteric microbiota presents a therapeutic target for reducing the severity of chemotherapy-associated mucositis.

## Results

### Doxorubicin induces aISC loss in GF mice

**Figure 1.**
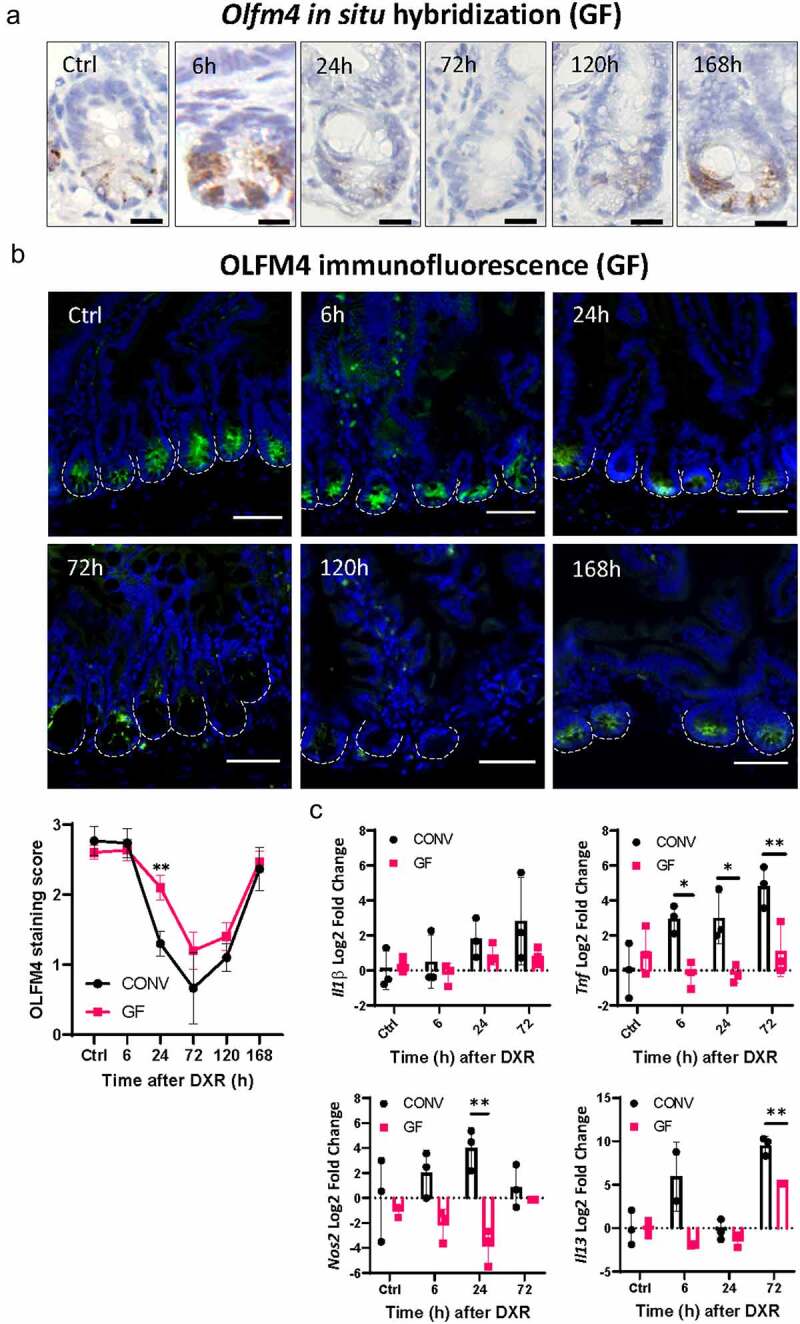
Doxorubicin induces active intestinal stem cell (aISC) loss in germ free (GF) mice.

We previously demonstrated that the subsequent immune cell infiltration and epithelial damage is dependent on the presence of enteric bacteria after DXR.^4^ Thus, we hypothesized that aISCs would be retained in GF mice, as they exhibit minimal immune cell infiltration and secondary tissue damage. We collected tissues from GF mice at various time points after DXR and performed OLFM4 immunostaining and *Olfm4 in situ* hybridization to label aISCs. Unexpectedly, we found that aISCs were reduced in GF conditions from 24 to 120 hours (5 days) after DXR, with a nadir at 72 hours (Figure 1a, 1b). aISCs, identified by OLFM4 immunostaining and Olfm4 in situ hybridization, returned by 168 hours (7 days) after DXR. This is a similar trend of aISC loss and recovery as in CONV mice, although GF mice exhibit less severe aISC loss at 24 hours post DXR (Figure 1b). DXR did not result in upregulation of pro-inflammatory gene transcripts *Tnf*α, *Il1β*, and *Nos2* in aISC depleted GF mice as compared to CONV mice (Figure 1c). Increased Il13 was observed in GF mice 72 hours post DXR, although significantly less than the upregulation observed in CONV mice (Figure 1c). These data suggest that aISC depletion in GF mice is unlikely to be due to TNF or IL1β-driven pro-inflammatory responses within the intestine. Secretion of IL-13 from crypt-adjacent group 2 innate lymphoid cells may be important in the promotion of ISC recovery from DXR and at least partially independent from the influence of the enteric microbiota.^15^

(A) Representative images of *Olfm4 in situ* hybridization in jejunal crypts over time (indicated in hours) after doxorubicin (DXR) injection. Representative of *n* = 3 animals/timepoint. Scale bar 10 μm. (B) Representative images and quantification of OLFM4 immunofluorescence in jejunal tissue over time (indicated in hours) after DXR. Crypts are outlined by the white dotted lines. Scale bar 50 μm. See methods for staining rubric. Data are presented as mean ± SD (n = 3 animals/timepoint). ** *p* < .01; two-way ANOVA followed by Tukey’s HSD post hoc test. (C) Log2 fold change of *Il1β*, Tnf, Nos2, and Il13 normalized to *Actb* in jejunal tissue at selected timepoints after DXR in CONV and GF mice. Data are presented as mean ± SD. * *p* < .05; ** *p* < .01; two way ANOVA followed by Tukey’s *post hoc* test.

### AMBx mice are protected from DXR-induced aISC loss

**Figure 2.**
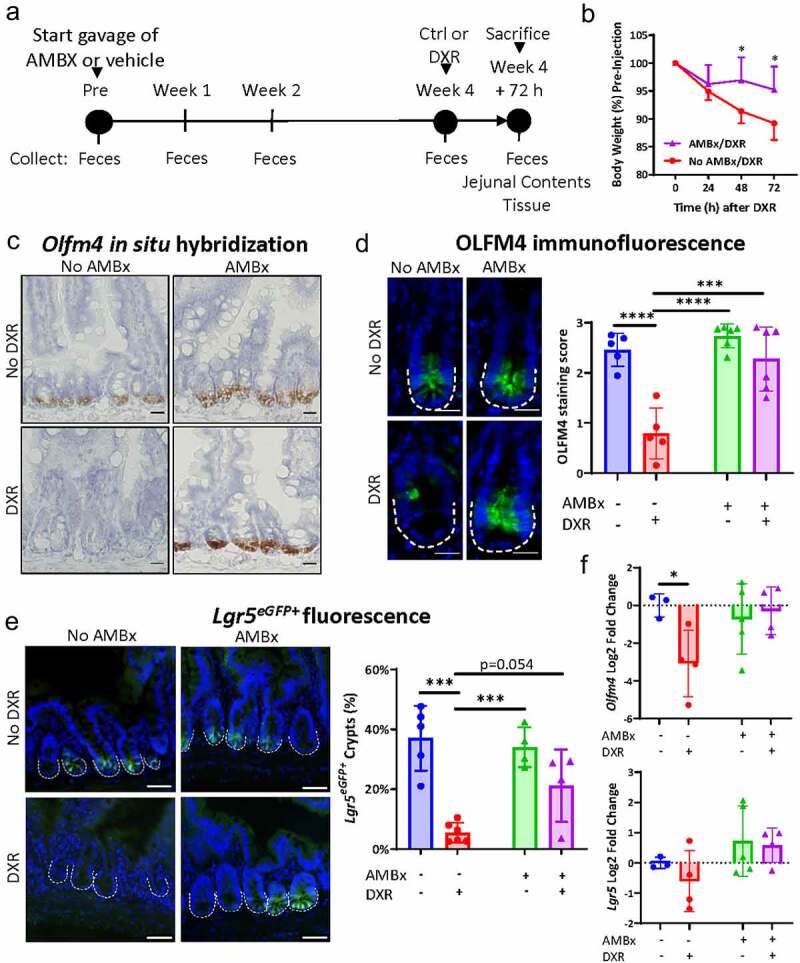
Antimicrobial-treated mice retain aISCs after DXR.

We then determined the effect of AMBx on aISCs after DXR. We monitored aISC presence by assessing the retention of two stem cell markers: Lgr5 and Olfm4. Similar to our previous paper we administered broad-spectrum oral antimicrobials then injected with DXR or no treatment.^5^ However, we modified our previous protocol to be administered by twice daily oral gavage of the antimicrobial solution to minimize variability of ingested drug and to avoid any influence from added sweetener in the solution.^13^ Control mice (non-AMBx) were gavaged with the same volume of vehicle used to suspend the antimicrobials (water) to account for any influence from the minor procedural stress of gavage. We collected all tissues at 3 days (72 hours) post DXR as the maximal depletion of aISCs is observed by this time point post DXR in GF (Figure 1b) and CONV mice (Figure 2a).^6^

(A) Experimental design to examine the influence of prolonged antimicrobial treatment (AMBx) delivered via gavage versus non-AMBx mice gavaged with water (vehicle). Feces were collected as indicated. At the end of 4 weeks, mice were either injected with DXR or no treatment and sacrificed 3 days later. (B) Weight loss over time after DXR injection in the two DXR-treated groups. Data are presented as mean ± SD (*n* = 8/group). * *p* < .05; repeated measures two way ANOVA followed by Holm-Sidak*post hoc* test. (C) Representative images of *Olfm4 in situ* hybridization in the indicated experimental groups. Scale bar 20 μm. Representative of *n* = 3–5 animals/group. (D) Representative images and quantification of OLFM4 immunofluorescence in the indicated experimental groups. Crypt bases are outlined by the white dotted lines. See methods for staining rubric. Scale bar 20 μm. Data are presented as mean ± SD. *** *p* < .001; **** *p* < .0001; two-way ANOVA followed by Tukey’s HSD *post hoc* test. (E) Representative images and quantification of Lgr5^eGFP+^ fluorescence in the jejunum of *Lgr5^eGFP-CreERT^^2^* mice in the indicated experimental groups. Crypt bases are outlined by the white dotted lines. Scale bar 50 μm. Data are presented as mean ± SD. *** *p* < .001; **** *p* < .0001; two way ANOVA followed by Tukey’s HSD *post hoc* test. (F) Log2 fold change of *Olfm4* and *Lgr5* expression normalized to *Actb* in jejunal tissue in the indicated experimental groups. Data are presented as mean ± SD. * *p* < .05; two way ANOVA followed by Tukey’s HSD *post hoc* test.

AMBx-treated mice lost significantly less weight than non-AMBx mice over time (Figure 2b). *Olfm4 in situ* hybridization demonstrated retention of *Olfm4* expression after DXR within the crypt base of AMBx mice (Figure 2c), consistent with the persistent OLFM4 immunofluorescence in AMBx mice after DXR (Figure 2d). A subset of the experimental mice were *Lgr5^eGFP-CreERT^^2^* mice, allowing for assessment of aISCs via green fluorescent protein (GFP) expression (Figure 2e). The *Lgr5^eGFP-CreERT^^2^* mouse exhibits mosaicism, resulting in fluorescence in about 30% of jejunal crypts.^2,16^ AMBx mice post DXR did not lose aISCs as GFP+ crypts were retained (mean 21.2 ± 12.1% crypts) vs non-AMBx mice treated with DXR (mean 5.5 ± 3.3% crypts) (Figure 2e). Consistent with these data, *Lgr5* and *Olfm4* transcripts in jejunal tissues did not decrease in AMBx mice after DXR (figure 2f), whereas non-AMBx mice after DXR had decreased transcripts of *Lgr5* and *Olfm4* (figure 2f). These data demonstrate that AMBx treatment is associated with aISC retention in the face of DXR-induced epithelial damage.

### AMBx mice have reduced pro-inflammatory transcripts and immune cell infiltration after DXR

We have previously shown that inflammatory cell infiltrate which appears in non-AMBx mice 5 days post-DXR is abrogated in AMBx mice.^5^ We considered that upregulated pro-inflammatory transcripts and the presence of inflammatory cell infiltrate could be transiently present at 3 days in AMBx mice. Therefore, we next evaluated DXR-induction of pro-inflammatory gene expression and lamina propria macrophage infiltration in AMBx mice compared to non-AMBx mice.

**Figure 3.**
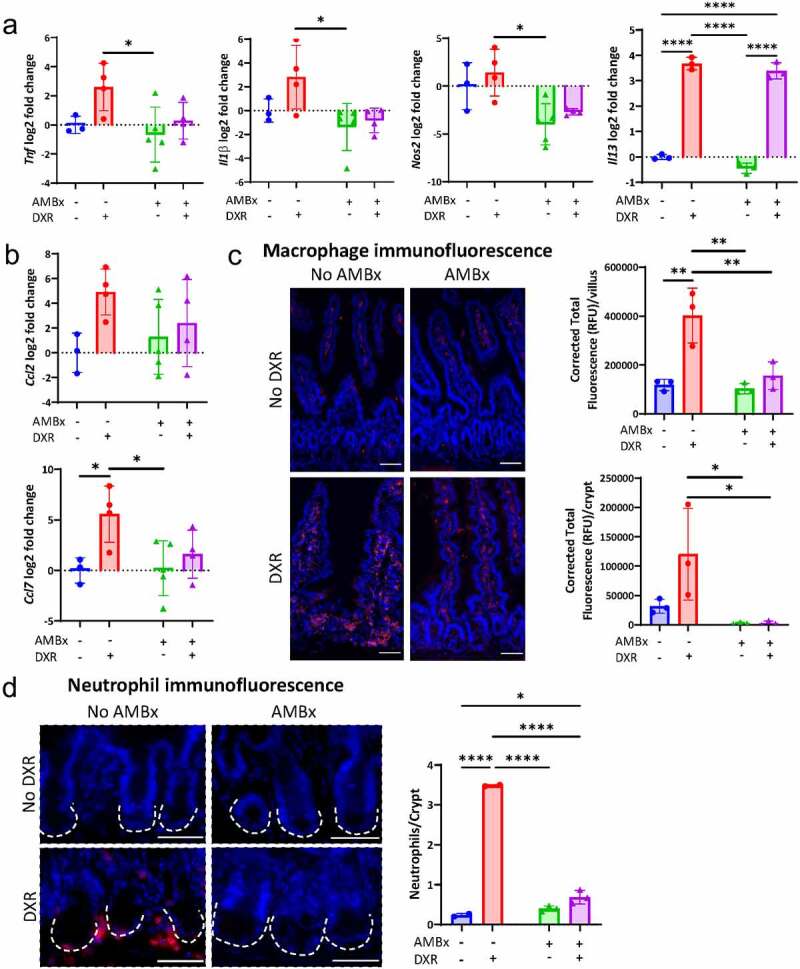
DXR-induced pro-inflammatory transcripts and immune cell infiltration are abrogated by pre-treatment with AMBx.

Pro-inflammatory transcripts *Tnfα, Il1β*, and *Nos2* were variably upregulated after DXR in non-AMBx mice, but not in AMBx mice (Figure 3a). These data suggest that AMBx mice do not respond to DXR-induced damage with increased expression of pro-inflammatory transcripts, presumably due to the influence of an altered microbiota. Both DXR treated groups exhibited upregulation of I113, suggesting that IL-13 may be produced within the intestinal tissues in response to DXR damage, regardless of aISC loss or inflammation (Figure 3a). This is consistent with the upregulation of Il13 also observed in GF mice after DXR (Figure 1c). The chemokine transcripts *Ccl2* and *Ccl7* were upregulated after DXR in non-AMBx mice (Figure 3b). There was more variation in these chemokine transcripts in the AMBx mice after DXR, suggesting there could be potential recruitment of inflammatory cells such as macrophages and neutrophils into the tissues of AMBx mice. Although there is no direct link between these immune cells and aISCs, they produce tissue-damaging products, such as reactive oxygen species (ROS) as part of their defense mechanisms. Therefore, we assessed macrophage and neutrophil infiltration by immunofluorescence of the jejunal tissues collected from all experimental groups.

(A) Log2 fold change of *Tnfα, Il1β Nos2, and Il13* expression normalized to *Actb* in jejunal tissue in the indicated experimental groups. Data are presented as mean ± SD. * *p* < .05; **** *p* < .0001; two way ANOVA followed by Tukey’s HSD *post hoc* test. (B) Log2 fold change of *Ccl2* and *Ccl7* expression normalized to *Actb* in jejunal tissue in the indicated experimental groups. Data are presented as mean ± SD. * *p* < .05; two way ANOVA followed by Tukey’s HSD *post hoc* test. (C) Representative images and quantification of macrophage infiltration into jejunal tissues identified by F4/80 immunofluorescence in the indicated experimental groups. Scale bar 50 μm. Data are presented as mean ± SD. Villus and crypt F4/80 fluorescence was calculated by measuring the fluorescent signal within the lamina propria of the villus and surrounding the crypt base, respectively, and correcting for background epithelial cell fluorescence (n = 10/animal). * *p* < .05; ** *p* < .01; two way ANOVA followed by Tukey’s HSD post hoc test. (D) Representative images and quantification of neutrophil infiltration adjacent to jejunal crypts identified by immunofluorescence in the indicated experimental groups. Neutrophils were quantified by counting the number of neutrophils adjacent to a crypt (outlined by the white dotted lines) (n = 10/animal). Scale bar 50 μm. Data are presented as mean ± SD. * *p* < .05; **** *p* < .0001; two way ANOVA followed by Tukey’s HSD post hoc test.

Non-AMBx mice after DXR exhibited macrophage infiltration into the lamina propria of the jejunum (Figure 3c, bottom left). These macrophages were broadly present throughout the lamina propria, not restricted to the crypt base, which suggests they are responding to non-localized chemotaxis signals along the crypt-villus axis (Figure 3c). Tissue macrophages are normally observed in homeostasis, as evident in the villi of both non-DXR groups (Figure 3c, top). However, after DXR, no increase in villus infiltration was observed in AMBx mice (Figure 3c, bottom right). Similar to macrophage infiltration, neutrophil presence was only observed in the non-AMBx DXR treated mice (Figure 3d, bottom right). Neutrophils, unlike macrophages, were only observed adjacent to the crypt, and were not observed in non-DXR tissues (Figure 3c). Neutrophils form the primary cellular infiltrate in crypt abscesses in many enteric diseases (Mathews 2014 Toxicologic Biology).^17^ These data suggest that AMBx mice lack macrophage and neutrophil infiltration subsequent to DXR, presumably due to a lack of immune stimulation derived from the enteric microbiota. Additionally, this absence of immune infiltration is linked to the secondary tissue damage that occurs from inflammatory cell products, including ROS.

### AMBx mice have increased epithelial mass and maintain crypt proliferative activity in vivo after DXR

We observed differences between GF and AMBx treated mice in stem cell preservation, therefore we considered that there may be a pro-proliferative response in AMBx mice. Increased proliferation has been associated with enhanced stem cell recovery after intestinal damage.^18^ Previous studies have shown that AMBx-treated mice have larger ceca and increased transcriptional expression of cell proliferation pathways in cecal enterocytes.^14^ Therefore, we examined the jejunal histomorphometry and proliferation in the same experimental groups as set forth in Figure 2a.

**Figure 4.**
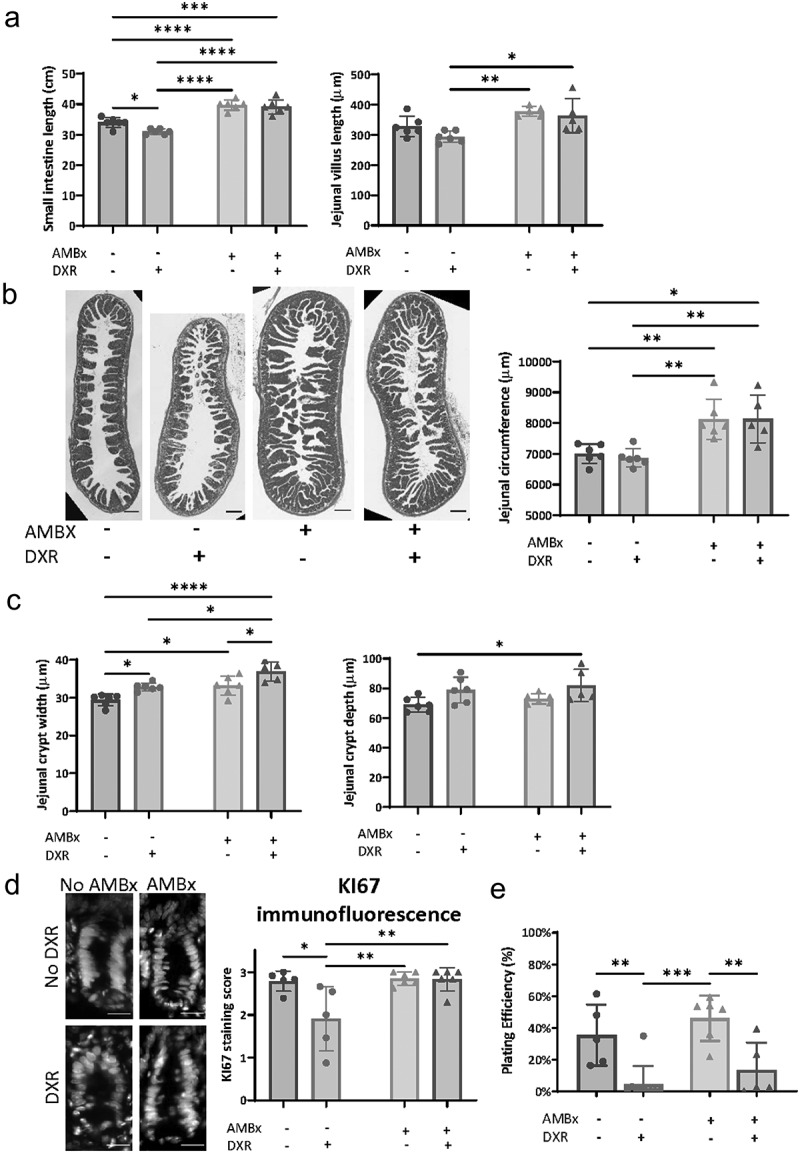
AMBX mice exhibit increased epithelial proliferation.

The small intestinal lengths of all AMBx mice were significantly longer than non-AMBx mice (Figure 4a). Additionally, the jejunal villi were longer in AMBx mice (Figure 4a) and significant jejunal dilation was observed in AMBx mice (Figure 4b). Taken together, these data suggest that AMBx treatment increased total epithelial mass in the small intestine. Despite the increased diameter and length of the small intestine in AMBx mice, there were limited increases in crypt width and depth (Figure 4c), suggesting that the increased epithelial mass was also associated with more total crypts/intestine. Cecal length was also increased in AMBx mice (Supplemental Figure 1), as demonstrated previously.^14^ DXR induced small intestinal shortening in non-AMBx mice (Figure 4a) which is consistent with the moderate to severe crypt loss typically observed in this model.^1^ AMBx mice were protected from this small intestinal shortening. We also evaluated cellular proliferation within the crypt epithelium using Ki67 immunofluorescence, a marker of proliferation that is present during all active phases of the cell cycle. No difference was observed between AMBx and non-AMBx mice without DXR treatment (Figure 4d). However, AMBx mice were protected from the impaired proliferation observed in non-AMBx mice after DXR treatment (Figure 4d).

(A) Total length of the small intestine measured at tissue collection in indicated experimental groups. Average villus length per animal (*n* = 30 villi/animal) in the indicated experimental groups measured on jejunal cross sections. (B) Representative images of cross sectional jejunal tissues stained with hematoxylin & eosin from the indicated experimental groups. Scale bar 200 μm. Average jejunal circumference measured on cross section images (*n* = 3–5 sections/animal). (C) Average jejunal crypt depth and width per animal (*n* = 30 crypts/animal) in the indicated experimental groups. (D) Representative crypt images of Ki67 immunofluorescence in the indicated experimental groups. Scale bar 20 μm. See methods for scoring rubric. (E) Organoid plating efficiency (% crypts growing 1 day after plating/total crypts plated) in the indicated experimental groups. For all panels, letters indicate groups that are significantly different from each other (*p* < .05, two way ANOVA followed by Tukey’s HSD *post hoc* test).

In spite of the increased proliferation and the retention of aISCs in DXR-treated AMBx mice, we found that isolated crypts from AMBx mice after DXR treatment grew poorly (Figure 4e). Regardless of AMBx treatment, DXR administration impaired the ability of crypts to grow in culture. Possible explanations for impaired *in vitro* growth include: 1) the stresses of isolation and culture on DXR-injured cells negates the enhanced survival ability of AMBx-treated aISCs, and 2) a pro-survival influence on the aISCs, such as microbial signaling, has been removed by transferring to culture.

### Antimicrobial administration induces shifts in the fecal microbiota over time

Given the differences between GF and AMBx mice, we examined the fecal microbiota alterations induced by AMBx treatment. We monitored fecal bacterial load and microbiota composition over time after initiation of AMBx and after DXR. Samples were obtained as indicated in Figure 2a to monitor the bacterial composition of the feces and the jejunal contents.

**Figure 5.**
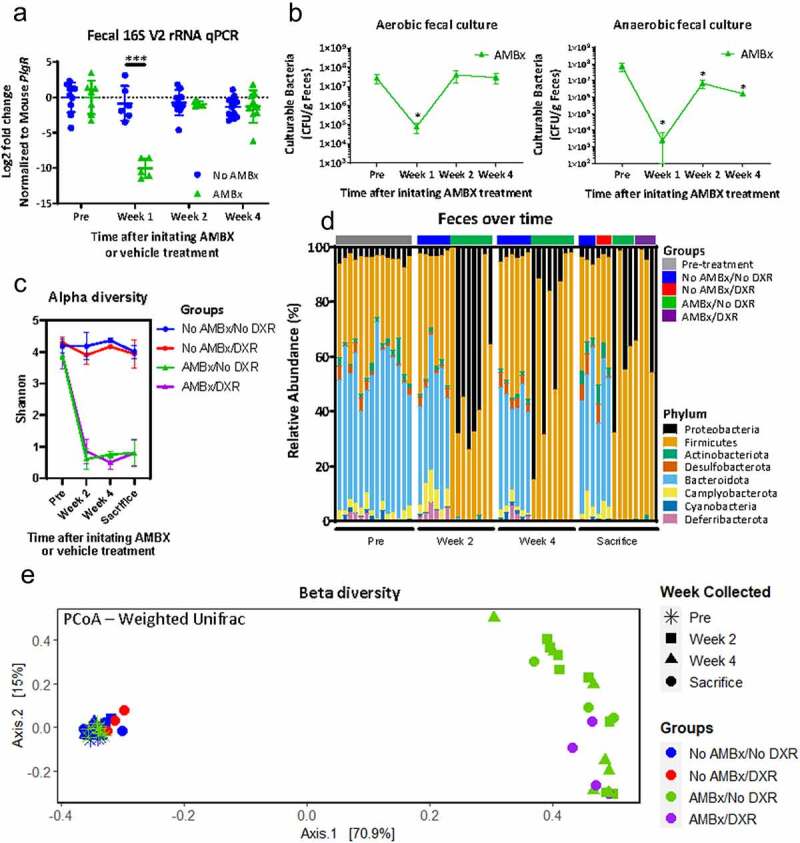
AMBX treated mice exhibit altered fecal microbiota.

Previous studies have indicated a depletion of the microbial load after a similar AMBx treatment.^13,14^ We identified a significant but transient depletion of total bacteria at 1 week post initiation of AMBx (Figure 5a, 5b). However, in subsequent weeks, the total bacterial load rebounded to similar levels as pre-AMBx, albeit with a modest reduction in culturable anaerobic fecal bacteria still present (Figure 5a, 5b). Visual inspection of the culture plates demonstrated that AMBx-treated culture plates exhibited a morphologically uniform appearance of the majority of the colonies. This was supportive of an altered microbiota with decreased diversity in the AMBx mice.

(A) Log2 fold change of the V2 region of 16S rRNA isolated from feces obtained from mice treated with AMBx or vehicle control as per Figure 2a. 16S rRNA signal was normalized to host DNA (mouse *PlgR*). **** *p* < .0001; mixed effects model followed by Holm-Sidak*post hoc* test. (B) Aerobic and anaerobic bacterial enumeration on feces acquired from AMBx mice. * *p* < .05; repeated measures one way ANOVA followed by Holm-Sidak*post hoc* test. (C) Shannon alpha diversity of fecal bacteria over the study period. *n* = 3–4 animals/group. (D) Relative abundance of bacterial phyla in the feces of each animal in the indicated groups over the study period. (E) Principal coordinates analysis (PCoA) derived from weighted UniFrac distances among fecal samples of the four experimental groups over time. For each axis, the percent of variation explained is reported in square brackets. Only feces obtained after DXR administration are identified as DXR in the Groups legend and on the plot (purple).

We performed 16S rRNA sequencing to further investigate the apparent alterations in microbial diversity induced by AMBx treatment. We identified a loss of species diversity in the feces in AMBx-treated mice (Figure 5c). This decrease in species diversity persisted throughout the AMBx treatment, suggesting that despite the increased bacterial load at later time points, the fecal bacterial diversity did not increase. An expansion of the Proteobacteria and Firmicutes phyla was observed in all AMBx treated mice, with nearly complete depletion of Bacteroidota (Figure 5d). Similarly, Zarrinpar *et al* demonstrated that feces from mice treated with a nearly identical broad spectrum antibiotic regimen had expansion of Proteobacteria.^14^

We then examined the beta diversity of the samples, which is a measure of the similarities between microbial communities. A distance matrix was calculated using the phylogenetic distances between the identified operational taxonomic units (OTUs) in the samples, weighted by the abundance of these OTUs (weighted UniFrac distance). This matrix was visualized using a principal coordinates analysis (PCoA) ordination plot, where the similarity between sample points is represented by the distance between them: points close together are more similar in microbial community composition than those points that are far apart. The microbial communities observed after AMBx initiation were distinct from non-AMBx mice (Figure 5e), as expected from previous studies.^5,13,14^ All mice before treatment clustered together, and the microbial communities in non-AMBx mice did not undergo a shift over the experimental period (Figure 5e). This suggests that the gavage-related stress had minimal impact on microbial communities, thus the shift observed in AMBx mice over time was due to AMBx treatment. No consistent shift in fecal microbial communities was observed after DXR in either group, although any modest impact may have been obscured by individual variation and small sample size (Groups: No AMBx+DXR (red) vs AMBx+DXR (purple); Figure 5e). There were no apparent pre-DXR differences between the two AMBx-treated experimental groups in beta diversity (Supplemental Figure 2). These data demonstrate that AMBx treatment produces a dramatic shift in the fecal microbiota.

### Antimicrobial administration alters the local jejunal microbiota

**Figure 6.**
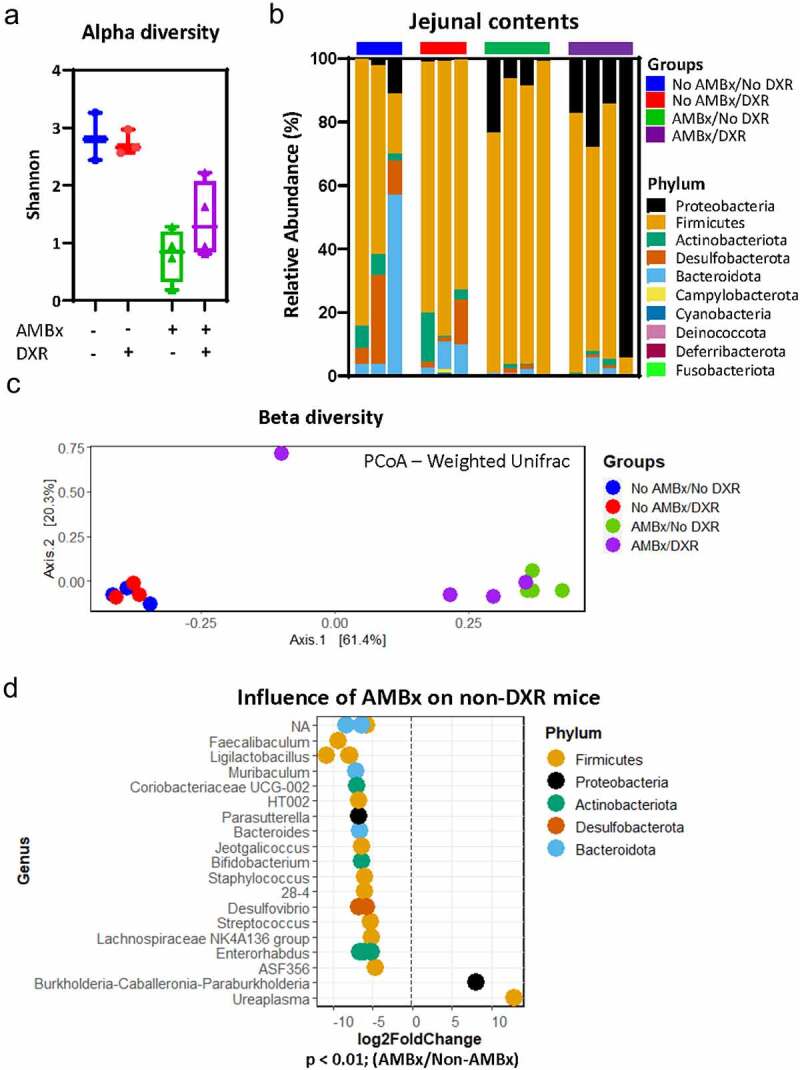
AMBx mice have an altered jejunal microbiota with significant expansion of *Ureaplasma* and *Burkholderia.*

We next examined the jejunal microbiota to understand local microbial alterations that may be directly impacting the aISCs. Due to the difficulty of obtaining this sample ante-mortem, all jejunal samples were obtained at the time of sacrifice from the lumen of the intestine (Figure 2a). Similar to the fecal samples, alpha diversity was reduced after AMBx treatment in the jejunal contents (Figure 6a). Consistent with the lower bacterial diversity present in the small intestine, the non-AMBx jejunal samples had modestly reduced levels of alpha diversity as compared to fecal samples (Figure 5c).

(A) Shannon alpha diversity of jejunal contents obtained from the experimental mice at the conclusion of the study period. Data are presented as box and whisker plots (min-max). (B) Relative abundance of bacterial phyla in the jejunal contents of each animal in the indicated groups at the conclusion of the study period. (C) Principal coordinates analysis (PCoA) derived from weighted UniFrac distances among jejunal samples of the four experimental groups collected at the conclusion of the study period. For each axis, the percent of variation explained is reported in square brackets. (D) Antimicrobial influences on the jejunal microbiota of non-DXR treated mice. The plot shows significantly different average log2 fold change of taxa abundance in AMBx mice as compared to non-AMBx mice. *p* < .01, Wald test followed by Benjamini-Hochberg correction for multiple comparisons (*n* = 3–4/group).

Jejunal contents in non-AMBx mice were largely composed of Firmicutes phylum, regardless of DXR treatment (Figure 6b). A modest expansion of Proteobacteria was observed in non-DXR-treated AMBx mice although the Firmicutes were still the most abundant phylum (Figure 6b). Interestingly, after DXR, Proteobacteria was the most abundant in one AMBx-treated animal (Figure 6b). In general, there was higher individual animal variation in relative phyla abundance in the jejunal contents as compared to the fecal samples (Figure 6b).

A principal coordinates of analysis (PCoA) plot performed with weighted UniFrac distances (taking into account the OTU abundance) was used to interrogate the jejunal beta diversity. The beta diversity was strongly influenced by the AMBx treatment, similar to the fecal communities (Figure 6c). There was no apparent impact by DXR treatment on the community beta diversity in 3 out of 4 jejunal samples. The AMBx and DXR-treated animal with a high abundance of Proteobacteria did exhibit a divergent community from the rest of the AMBx-treated samples.

We performed pairwise comparisons of the jejunal contents to determine significantly different OTUs between experimental groups. First, we examined the interaction between DXR and the microbiota by comparing AMBx to non-AMBx groups of DXR-treated mice. We found significantly increased OTUs within the phyla Proteobacteria (*Burkholderia, Pseudomonas*, and *Ochrobactrum*) and Firmicutes (*Streptococcus, Ureaplasma*) in DXR-treated AMBx jejunal contents when compared to DXR-treated non-AMBx mice (Supplemental Figure 3a). As *Ochrobactrum* was the only genus significantly increased in DXR-treated AMBx mice as compared to non-DXR AMBx mice (Supplemental Figure 3b), it appears that DXR treatment specifically in AMBx mice allowed for expansion of *Ochrobactrum* in jejunal contents. In non-AMBx animals, DXR treatment was associated with increases in several genera of the phylum Firmicutes (Supplemental Figure 3c).

We hypothesized that the microbiota present at the time of injection would be the most influential on the aISCs, as these cells are expulsed from the crypt base as early as 24 hours after DXR injection.^6^ Therefore, we focused on the influence of AMBx treatment in the jejunum. We compared the differential expression of OTUs between non-AMBx and AMBx mice, without the confounding influence of DXR (Figure 6d). Several OTUs were significantly decreased in the AMBx mice, as expected with reduced species diversity (Figure 6d) and similar to that observed when comparing the two DXR treated groups (Supplemental Figure 3c). Significantly increased OTUs in AMBx mice without DXR’s influence were identified in Firmicutes and Proteobacteria phyla, specificially genera *Burkholderia* and *Ureaplasma* (Figure 6d). These two genera were also increased in jejunal contents of AMBx mice 3 days after DXR administration (Supplemental Figure 3a). As these genera were identified in both groups of AMBx mice, and given that only AMBx treated mice retain aISCs after DXR, we suggest that these bacteria may have a pro-survival influence on aISCs. Future studies examining the potential influence of these taxa may illuminate therapeutic targets for enhancing aISC survival after injury.

## Discussion

In this study, we have demonstrated that AMBx pre-treatment alters the jejunal microbiota and retains the aISCs in the face of DXR-induced damage. We have previously shown that the subsequent pro-inflammatory response and immune cell infiltration is dependent on the presence of enteric bacteria.^5^ We confirmed this finding in the current study in addition to demonstrating that GF mice have reduced aISC markers due to DXR. Based on the unique retention of aISCs in the AMBx model, we demonstrate here that jejunal microbiota present after AMBx treatment influence aISC behavior in response to chemotherapy.

Using a similar AMBx protocol, Zarrinpar *et al* demonstrated that AMBx treated mice exhibited an elongated and enlarged intestine, consistent with our findings.^14^ Cecal tissues isolated from AMBx mice expressed increased glucagon-like peptide-1 (*Glp-1*), an incretin involved with management of the enteroinsular axis and proliferation.^14^ Additionally, AMBx treatment altered bile acid metabolism and cellular metabolism within the cecal enterocytes, though it is unclear whether these alterations extend to the small intestine.^14^ There are distinct metabolic differences between the small intestinal epithelium and the cecal and colonic epithelium.^19^ Therefore, Zarrinpar *et al*’s findings in the cecal tissue cannot be directly applied to the small intestine.^14^ We have been unsuccessful in identifying enhanced aISC survival after DXR in mice treated with a GLP-1 agonist.

The differences between jejunal and fecal bacterial composition identified here support the importance of examining the microbiota associated with the region of interest.^20^ AMBx resulted in a general decrease in the majority of intestinal microbes, as expected due to the antimicrobial administration. Within the jejunal microbiota examined here, we found significant expansion of two genera in AMBx treated mice, *Burkholderia* and *Ureaplasma*. Members of both genera have been associated with antimicrobial resistance and opportunistic infections in immunocompromised patients.^21,22^ AMBx depletion of susceptible bacteria likely allowed for the expansion of these antimicrobial resistant taxa within the jejunal contents.

It is unknown whether these identified genera are a causal factor in the retention of aISCs in this model. It is possible that one or both of the identified genera manipulates the balance of apoptotic vs pro-survival signaling within the crypt. As mentioned previously, LPS stimulation of TLR4 causes aISC apoptosis,^12^ whereas bacterial-derived lactate and stimulation of NOD2 by intracellular MDP are cytoprotective for aISCs.^7,9,10^ Enteroid yield was enhanced by MDP stimulation of enteroids isolated from mice 72 hours after DXR, suggesting that MDP stimulation is important for aISC survival after DXR.^10^ MDP is derived from peptidoglycan from both gram positive and negative bacteria, thus both genera could stimulate NOD2. *Burkholderia* spp. express LPS although the pathogenicity varies between species, depending on the lipid A structure.^23^ Thus, this genera could negatively impact aISCs through TLR4 signaling. In contrast, *Ureaplasma* spp. lack LPS and suppress TLR4 expression in monocytes.^24^ Additionally, *Ureaplasma* spp. suppress inflammatory forms of cellular death (such as pyroptosis) in endothelial cells.^25^ Therefore, it is possible that *Ureaplasma* in particular could drive microbiota-host signaling toward an aISC-protective environment. Further investigation of the epithelial host response to these two genera is required to determine whether they are associated with the retention of aISCs in this model. Approaches such as fluorescence *in situ* hybridization with taxa-specific probes may identify proximity of these bacteria to aISCs at the base of the intestinal crypts. Alternatively, co-culture of organoids with these specific genera could reveal a direct communication with epithelial cells that enhances aISC survival. If identified, this would also support a host-microbiota relationship underpinning aISC survival in this model.

DXR is a chemotherapeutic anthracycline produced by a strain of *Streptomyces peucetius*. DXR has antibiotic activity via induction of DNA damage in bacteria, and thus could have an impact on the intestinal microbiota. We identified several taxa that are modestly affected by DXR injection, although it is unclear whether this is a direct or indirect (secondary to host response) function of DXR treatment. In addition, DXR can be inactivated via deglycosylation by environmental Actinobacteriota,^26^ and by a variety of Enterobacteriaceace found in the human gut.^27^ We did not identify expansion of Actinobacteriota in AMBx treated mice, but there was a modest increase in Proteobacteria (which include the Enterobacteriaceace family). The identified primary driver of the Proteobacteria expansion in AMBx treated mice, *Burkholderia*, is not in the Enterobacteriaceace family. It is unknown whether they may share the ability to detoxify DXR. Thus, alterations in microbiota could alter the concentration of active DXR available to induce intestinal epithelial damage.

We believe that results from AMBx mice are more reflective of clinically relevant host responses to intestinal damage. GF mice have an altered development of the immune system, including intestinal innate immunity, and the enteric nervous system.^28^ Broad spectrum AMBx administered to adult mice has an advantage over use of GF mice, as AMBx mice have normal growth and development associated with microbial signaling prior to microbial depletion.^13^ The ability to manipulate aISC survival or apoptosis via microbial signaling has broad ranging implications for intestinal regenerative therapies and cancer biology.

## Methods

### Mice

C57Bl6/J mice, bred in-house at North Carolina State University, were used for the majority of experiments, except for where indicated. *Lgr5^eGFP-CreERT^^2^* mice were bred in-house at North Carolina State University from established lines (all on C57Bl6/J background). Gnotobiotic (germ free) C57Bl6 mice were managed and treated with DXR by the Gnotobiotic Core at North Carolina State University. All animals were cared for under the North Carolina State University’s IACUC guidelines. Experiments were performed by B. Sheahan.

All experiments were performed on 12–24 week old mice, with approximately equal numbers of male and female mice. Prior to receiving antimicrobials, all animals were co-housed with same-sex litter mates, which were used as controls whenever possible. The *n* for each experiment is indicated either in the figure legend or graphically represented by symbols in the figure panel.

### Antimicrobial administration

Mice were administered a broad-spectrum mixture of antimicrobials or sterile water for four weeks modified from Reikvam *et al*.^13^ Mice were administered amphotericin B (1 mg/kg) for 3 days of twice daily gavage to prevent development of mycotic typhlitis. The amphotericin B was diluted in sterile water to administer 10 µl/1 gram BW. Following the 3 days of antifungal, mice were gavaged twice daily with the following mixture: 1 mg/kg amphotericin B, vancomycin 50 mg/kg, metronidazole 100 mg/kg, and neomycin 50 mg/kg, dissolved in sterile water to administer a volume of 10 µl/gram BW. Antimicrobial treated mice also received 1 mg/1 mL of ampicillin dissolved in drinking water. Control mice were gavaged with sterile water (10 µl/1 gram BW) twice daily to keep the gavage-induced stress consistent between groups. Mice were singly housed for 5–7 days prior to initiating the treatment to allow acclimatization, then kept singly housed for the duration of the treatment. Gavage treatments were continued until sacrifice.

### Doxorubicin injection

Mice were injected once with 20 mg/kg doxorubicin HCl (Actavis Pharma, New Jersey, USA) intraperitoneally four weeks after initiation of gavage with antimicrobials, as previously reported.^1,5^ Mice were monitored for weight loss daily. If mice lost >20% of initial body weight, they were euthanized in accordance with IACUC protocol.

### Isolation of jejunum

Mice were euthanized by cervical dislocation after anesthesia with isoflurane. Small intestine was immediately collected after euthanasia and flushed with ice-cold 1x PBS (Ca^2+^ and Mg^2+^ free) except for when jejunal contents were collected for sequencing. The small intestine proximal to the ligament of Treitz (signifying the duodenojejunal juncture) was discarded, and the proximal one-half (approximately 10–12 cm) of the remaining intestine was identified as jejunum.

### Quantitative reverse-transcriptase PCR (qRT-PCR)

Total RNA was isolated from jejunal tissues with the RNeasy Mini kit (Qiagen) per the manufacturer’s protocol. Quality of mRNA was verified with a Nanodrop 2000 spectrophotometer (Thermo Fisher). 500 ng cDNA was synthesized using the High-Capacity cDNA Reverse Transcription Kit (Applied Biosystems), with RNase A included in the reaction, following the manufacturer’s protocol. qRT-PCR analysis was performed with 25 ng cDNA/well using Taqman Universal Master Mix II with UNG (Applied Biosystems), on a QuantStudio 6 PCR system (Thermo Fisher) for the following Taqman probes: *Actb* (Mm02619580_g1), *Lgr5* (Mm00438890_m1), *Olfm4* (Mm01320260_m1), *Tnf* (Mm00443258_m1), *Il1β* (Mm00434228_m1), *Nos2* (Mm00440502_m1), *Il13* (Mm00434204_m1), *Ccl2* (Mm00441242_m1), *Ccl7* (Mm00443113_m1). Signals were normalized to *Actb* for each sample, and relative fold changes were calculated via ΔΔCt analysis.

### Jejunal crypt isolation

Chemical and mechanical dissociation was performed to obtain jejunal crypts as previously described with modifications.^29^ After filleting the length of the isolated jejunum, the jejunum was incubated in 30 mM EDTA (pH 7.4) for 30 minutes on ice. Tissue was transferred to 1x PBS (Ca^2+^ and Mg^2+^ free), and mechanical dissociation (shaking) was performed to exfoliate crypts from the underlying lamina propria. Crypts were separated from intact villi by passage through a 70 um cell strainer prior to counting.

### Crypt culture

Approximately 100 isolated jejunal crypts were resuspended in 20 μl Matrigel (Corning) and placed in 48 well tissue culture plates. After polymerization of the Matrigel, 250 μl of media was added per well. The media consisted of Advanced DMEM/F12 (Invitrogen) containing growth factors (50 ng/ml recombinant mouse EGF (R&D Systems), 500 ng/ml R-spondin 1 (R&D Systems), 100 ng/ml recombinant mouse Noggin (Peprotech), 1x N2 supplement (Gibco), 1x B27 (Gibco), 10 µM HEPES (Gibco), 1x Glutamax (Gibco), and 500 µg/ml penicillin-streptomycin (Gibco)). Media was changed every other day. Enteroids were imaged and counted one day after plating to determine % plating efficiency (# enteroids growing/#crypts plated) using an inverted Olympus IX83 microscope.

### Histomorphometry

For histology, jejunal tissues were fixed in 10% zinc formalin for 12–18 hours, transferred to 70% ethanol for < 2 weeks, and embedded in paraffin. 5 μm sections were adhered to Superfrost Plus slides (Thermo Fisher, 4951PLUS4) and stained with hematoxylin & eosin by the NCSU Histology Core. At least 3 cross sections of jejunum were imaged with an inverted Olympus IX83 microscope using brightfield microscopy. 10 villi or crypts were counted per section per mouse. Circumference, villi length, crypt depth, and crypt width were measured using FIJI (ImageJ) by a blinded observer.^30^

### Immunofluorescence

For immunofluorescence, jejunal tissues were fixed in 4% paraformaldehyde for 12–18 hours, dehydrated in 30% sucrose for 24 hours, embedded in OCT (optimal cutting temperature compound), and stored at −80°C until sectioning. 7 μm sections were adhered to Superfrost Plus slides (Thermo Fisher, 4951PLUS4) and OCT was removed by immersion in 1X PBS after drying. Sections where GFP fluorescence was imaged were then directly mounted with Hard Set mounting medium with DAPI (Vector Laboratories, H-1500) and imaged with an inverted Olympus IX83 microscope. For antibody-tagged sections, staining was performed as described below before mounting as for the GFP fluorescence. Control sections were incubated with IgG of the same primary species or blocking solution (5% BSA or 10% FBS in 1x PBS) for all immunofluorescent experiments.

For OLFM4 and Ki67 immunofluorescence, sections were antigen retrieved in sodium citrate buffer (2.94 g sodium citrate, 300 μl Tween 80, pH 6) using a pressure cooker. Sections were blocked in 10% FBS in PBS for 1 hour at room temperature, incubated overnight at 4°C with primary antibodies, washed in 1x PBS, and incubated for 1 hour at room temperature with secondary antibodies. Sections were immersed in 1x PBS and mounted as described above. Antibodies included: rabbit anti-OLFM4 (1:500, 39141S) and rat anti-Ki67 eFluor660 (1:250, 50–5698-82). The secondary antibody was donkey anti-rabbit AF488 (1:500, A21206). Sections were immersed in 1x PBS and mounted/imaged as described above. Sections were pseudocolored during processing for improved distinction.

For F4/80 and neutrophil immunofluorescence, sections were lightly fixed and permeabilized with methanol/acetone (50%/50%) for 20 minutes at −20°C. After washing with 1x PBS, sections were blocked with 5% BSA in PBS for 1 hour at room temperature, incubated overnight at 4°C with primary antibody, rat anti-F4/80 (1:200, ab6640, Abcam, Cambridge, MA, USA) or rat anti-neutrophil (1:250, ab2557, Abcam, Cambridge, MA, USA), washed in 1x PBS, and incubated for 1 hour at room temperature with secondary antibody, goat anti-rat APC (1:500, A10540). Sections were immersed in 1x PBS and mounted/imaged as described above. Sections were pseudocolored during processing for improved distinction. For F4/80 macrophage quantification in ImageJ, regions of interest (ROIs) were drawn around the villus lamina propria or the crypt base (encompassing the crypt and the surrounding subepithelial cells) to measure the Integrated Density and Area. Background mean fluorescence was obtained from ROIs containing nearby epithelial cells without any F4/80 staining. The Corrected Total Fluorescence = Integrated Density – (Area of ROI x Background mean fluorescence). For neutrophil quantification, the number of neutrophils was quantified adjacent to each crypt (>10 crypts/sample).

### In situ hybridization

*In situ* hybridization for *Olfm4* probe was performed using RNAscope chromogenic assay 2.5, with *Ppib* as positive control probe and *Dapb* as negative control probe. RNAscope’s recommended scoring guideline for positive cells was applied to hybridized slides by a blinded observer. Only epithelial crypt cells were considered to be cells of interest.

### Image acquisition

Images were captured using an inverted fluorescence microscope (Olympus IX83, Tokyo, Japan) fitted with a monochrome digital camera (ORCA-flash 4.0, Hamamatsu, Japan) and color camera (DP26, Olympus, Tokyo, Japan). The objective lenses used were X10, X20 and X40 with numerical apertures of 0.3, 0.45 and 0.6, respectively (LUC Plan FLN, Olympus, Tokyo, Japan).

### OLFM4 scoring

OLFM4 is difficult to quantify precisely as a secreted protein. Therefore, a blinded scoring system was adapted from Besson *et al* to capture four categories of OLFM4 immunopositivity.^31^ These were scored by the intensity of the fluorescence, to approximate the number of cells positive for OLFM4. Only complete jejunal hemicrypts were scored (> 10 crypts/mouse). The scoring scheme is as follows: 0: negative; 1: 1–2 positive cells/faint; 2: 3–5 positive cells/moderate; 3: > 6 positive cells/intense.

### *Ki67* scoring

Ki67 typically marks many cells in the crypt and is often overlapping, making absolute quantification difficult. Therefore, Ki67 immunopositivity was quantified using the following scoring scheme: 0: <5 positive cells; 1: 5–10 positive cells; 2: 10–20 positive cells; 3: >20 positive cells. Only complete jejunal hemicrypts were scored (>10 crypts/mouse).

### Bacterial culture

Feces were collected directly from mice via defecation into a pre-weighed sterile microcentrifuge tube. The feces were diluted in PBS and vortexed to make a slurry. Serial dilutions were performed to out to 1 e − 8 and plated for enumeration on either 5% Columbia sheep’s blood agar (Thermo-Fisher) or anaerobic agar (Thermo-Fisher). The aerobic cultures were placed in a 37°C 5% CO_2_ incubator for 48 hours. The anaerobic cultures were placed in an anaerobic jar chamber (Mitsubishi Gas Chemical) with AnaeroPack (Mitsubishi Gas Chemical) and an oxygen indicator to ensure maintenance of anaerobic conditions for 72 hours (37°C incubator). All plates were quantified by identifying the dilution that had 30–100 individual bacterial colonies present per 20 μl droplet. The bacterial load was calculated after accounting for the initial fecal pellet weight.

## 16S sequencing

Feces were collected directly from mice via defecation into a sterile microcentrifuge tube. Jejunal samples for 16S sequencing were obtained by physically extruding luminal contents from the entire jejunum into a sterile microcentrifuge tube. After collection of samples, tubes were then placed in liquid nitrogen and kept at −80°C until analysis.

For the jejunal analysis and paired fecal analysis, the University of Michigan Microbiome Core completed 16S sequencing of the V4 region. Fecal pellets or jejunal contents were inserted into premade microbiota plates prior to shipment to the Core. Microbial DNA was extracted from murine feces and jejunal contents using the Qiagen MagAttract PowerMicrobiome DNA/RNA kit (Qiagen, catalog no. 27500-4-EP). Extracted DNA was then used to generate 16S rRNA libraries for community analysis. The process used for library generation has been previously described by Seekatz et al.^32^ Briefly, barcoded dual-index primers specific to the V4 region of the 16S rRNA gene amplify the DNA.^33^ PCR reactions are composed of 5 µL of 4 µM equimolar primer set, 0.15 µL of AccuPrime Taq DNA High Fidelity Polymerase, 2 µL of 10x AccuPrime PCR Buffer II (Thermo Fisher Scientific, catalog no. 12346094), 11.85 µL of PCR-grade water, and 1 µL of DNA template. The PCR conditions used consisted of 2 min at 95°C, followed by 30 cycles of 95°C for 20 s, 55°C for 15 s, and 72°C for 5 min, followed by 72°C for 10 min. Each PCR reaction was normalized using the SequalPrep Normalization Plate Kit (Thermo Fisher Scientific, catalog no. A1051001). The normalized reactions were pooled and quantified using the Kapa Biosystems Library qPCR MasterMix (ROX Low) Quantification kit for Illumina platforms (catalog no. KK4873). The Agilent Bioanalyzer was used to confirm the size of the amplicon library (~399 bp) using a high-sensitive DNA analysis kit (catalog no. 5067–4626). Pooled amplicon library was then sequenced on the Illumina MiSeq platform using the 500 cycle MiSeq V2 Reagent kit (catalog no. MS-102-2003) according to the manufacturer’s instructions with modifications of the primer set with custom read 1/read 2 and index primers added to the reagent cartridge. The “Preparing Libraries for Sequencing on the MiSeq” (part 15039740, Rev. D) protocol was used to prepare libraries with a final load concentration of 5.5 pM, spiked with 15% PhiX to create diversity within the run. FASTQ files were generated for paired-end reads.

### Microbiota analysis

All analysis was performed in Rstudio (version 1.3.959; R version 4.0.4), using the R package DADA2 (version 1.16.0)^34^ for quality control and trimming, and the R packages phyloseq (version 1.32.0)^35^ and DESeq2 (version 1.28.1)^36^ for analysis, and the R package ggplot2 (version 3.3.3)^37^ for graphical representation. Reads were assessed for quality control, trimmed to remove low quality sequences and primer sequences, and aligned to a 16S rRNA reference taxonomic database (Silva version 138.1).^38^ Silva v138.1 is based on the Genome Taxonomy Database (GTDB).^39^ All low prevalence phyla (present in 0–1 samples), taxa in the Order Chloroplast, and taxa in the Family Mitochondria were removed but no other preprocessing (rarefication or normalization) was performed prior to analysis.^40^ Chloroplast and Mitochondria taxa were filtered out as they likely represent plant sequences in the diet. Total reads obtained from feces and from jejunal contents are graphically represented in Supplemental Figure 4a and 4b. Alpha diversity was evaluated in all samples by Shannon diversity, which weights the number of bacterial species by their relative evenness. Beta diversity, or the similarity between samples, was evaluated with a weighted UniFrac metric to determine the distance between samples and Principal Coordinates Analysis (PCoA) ordination plot was used to visualize the data. AMBx treatment resulted in overabundance of a few phyla so the weighted UniFrac metric was used, which takes into account the abundance of the OTUs.

### Statistical analysis

All statistics and preparation of graphs were performed in GraphPad 8 (LaJolla, CA). FIJI was utilized for image analysis, counting of cells, and calculation of corrected total fluorescence where indicated (ImageJ). No *a priori* calculations were performed for sample size analysis. Normality was assessed by Shapiro-Wilk and Q-Q plots prior to parametric testing by Student’s *t* test or ANOVA followed by *post hoc* testing as appropriate for the number of groups. Repeated measures ANOVA was performed for groups that included the same mice that were sampled over time. When there were missing values, instead of a repeated measures ANOVA we analyzed the data by fitting a mixed model as implemented in GraphPad 8. This mixed model uses a compound symmetry covariance matrix, and is fit using Restricted Maximum Likelihood (REML). In the presence of random missing values, the results can be interpreted like a repeated measures ANOVA. Where this was used, the Geisser-Greenhouse correction was applied to correct for non-equal sphericity. DEseq2 fits a negative binomial generalized linear model to each OTU abundance and tests differential expression by calculating a *p*-value with a Wald test followed by Benjamini-Hochberg correction for multiple tests. In each pairwise comparison, the untreated (either No AMBx or No DXR) group was considered as the reference population for calculation of the log2 fold change.

## Supplementary Material

Supplemental Material

## Data Availability

The data that support the findings of this study are available from the corresponding author, [CMD], upon reasonable request. Sequencing information is available through BioProject ID PRJNA782475.
