# Supplementary figures and images for: Prolonged oral antimicrobial administration prevents doxorubicin-induced loss of active intestinal stem cells
Source: Gut Microbes. 2022 Jan 11;14(1):2018898. doi: 10.1080/19490976.2021.2018898 (PMC8757478; doi:10.1080/19490976.2021.2018898)

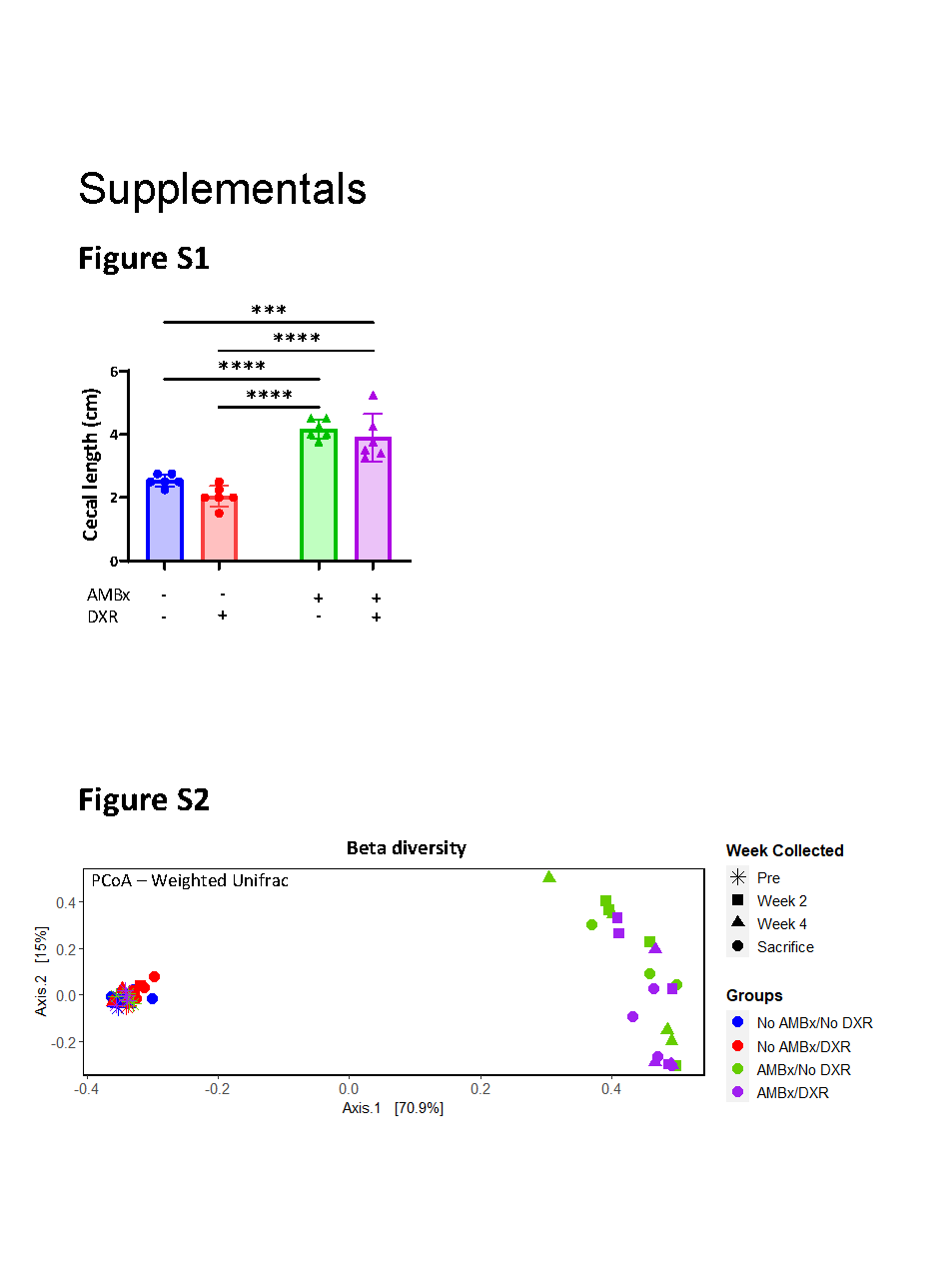

Supplement: Supplemental Material [file KGMI_A_2018898_SM5564.zip › supplementary/Supp Fig 1 and 2.tif]

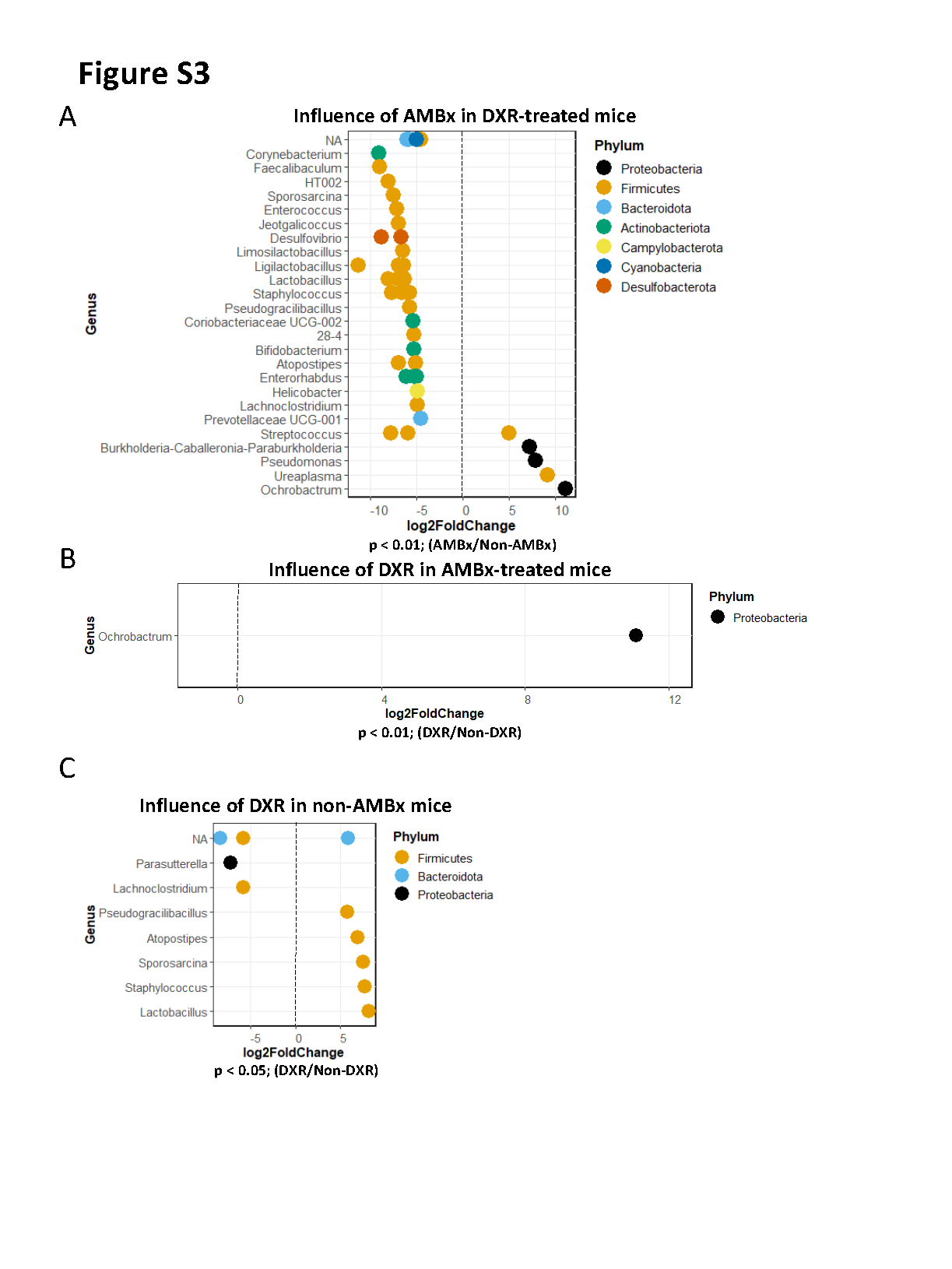

Supplement: Supplemental Material [file KGMI_A_2018898_SM5564.zip › supplementary/Suppl Fig 3.tif]

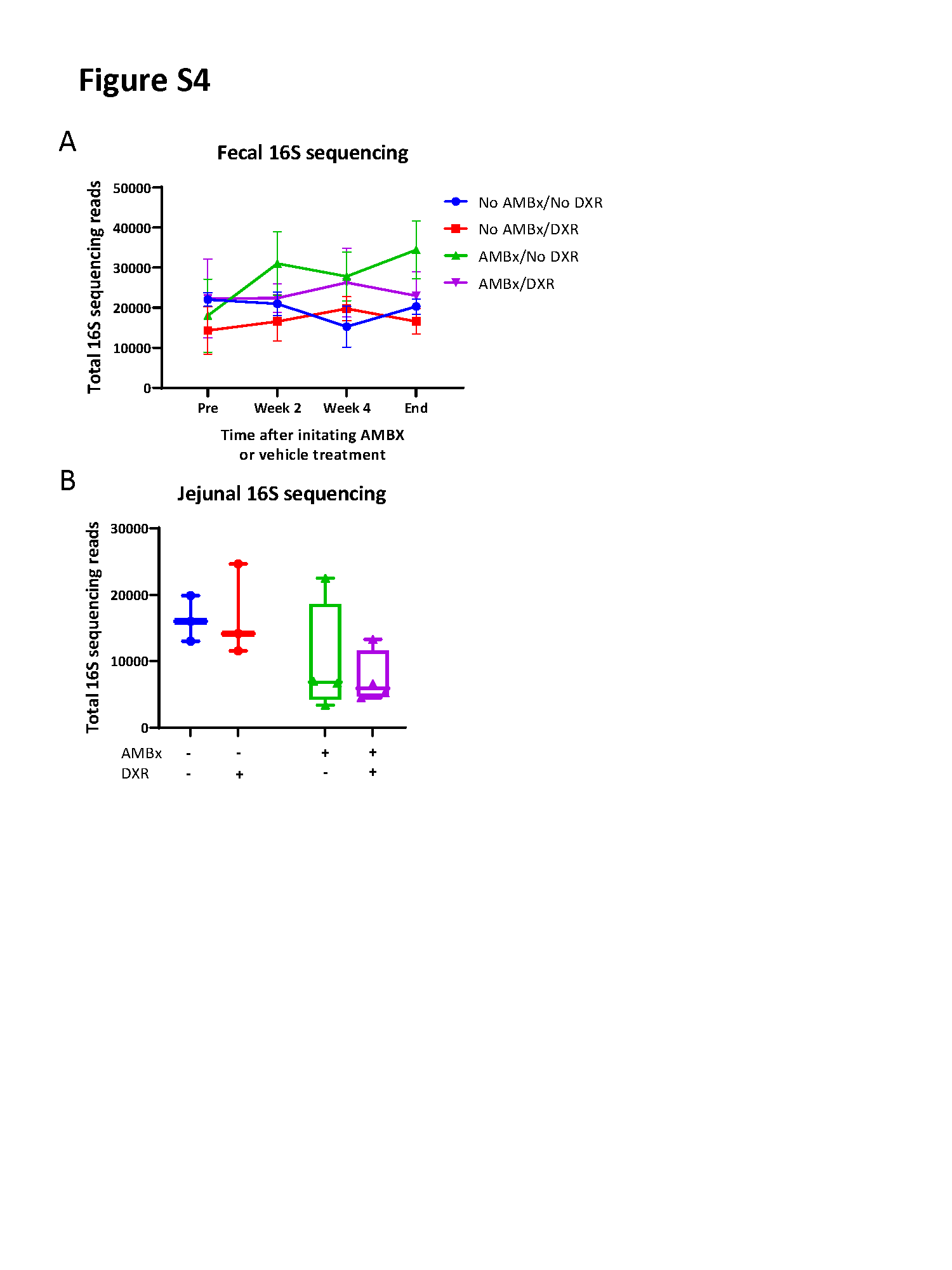

Supplement: Supplemental Material [file KGMI_A_2018898_SM5564.zip › supplementary/Suppl Fig 4.tif]
